# Anti-oxidant and anti-hyperlipidemic activity of *Hemidesmus indicus* in rats fed with high-fat diet

**Published:** 2016

**Authors:** Suganya Venkateshan, Vetriselvan Subramaniyan, Velmurugan Chinnasamy, Sarath Chandiran

**Affiliations:** 1 *Department of Pharmacology, Madras Medical College, Tamil Nadu, India*; 2 *Department of Medicine, Pharmacology Unit, College of Medicine and Health Sciences, Arba Minch University, Ethiopia *; 3 *Department of Pharmacology, Sri Krishna Chaithanya College of Pharmacy, Madanapalee, Andhra Pradesh, India *; 4 *Department of Pharmaceutical Sciences, Ratnam Institute of Pharmacy, Nellore, India*

**Keywords:** *Hemidesmus indicus*, *High fat diet*, *Oxidative stress*, *Plasma lipid profile*, *Antihyperlipidemia*, *Liver protection*

## Abstract

**Objective:**

Dietary changes play major risk roles in oxidative stress and cardiovascular disease and modulate normal metabolic function. The present study was designed to investigate the ameliorative potential of different extracts of *Hemidesmus indicus* to experimental high-fat diet in wistar rats, and their possible mechanism of action.

**Materials and Methods:**

Male wistar rats were divided into 6 groups (n=6/group) and fed with a standard diet (control), high-fat diet (HFD), high-fat diet supplemented with different extracts and positive control for 9 weeks. High-fat diet induced changes in average body weight and oxidative stress and elevated levels of plasma lipid profile in rats.

**Results:**

Oral administration of methanolic extract of *H. indicus *(200 mg/kg) offered a significant dose-dependent protection against HFD-induced oxidative stress, as reflected in the levels of catalase (p<0.001 in the aorta, heart and liver), superoxide dismutase (p<0.001 in the aorta, heart and liver), and glutathione peroxidase (p<0.001 in the aorta, heart and liver). Hyperlipidemia condition assessed in terms of body weight, total cholesterol, free cholesterol, ester cholesterol, phospholipids, triglycerides, and atherogenic index and the results showed significant differences between HFD and non-HFD fed rats (p<0.001). High-fat diet treated rats showed changes in hepatic tissue architecture such as micro and macrovascular steatosis, increased fatty infiltration, and inflammation.

**Conclusion:**

The present study revealed that the methanolic extract of *H. indicus *protects against oxidative stress, hyperlipidemia and liver damage.

## Introduction

Excess accumulation of body fat is one of the leading causes of death worldwide. The prevalence of metabolic diseases such as obesity and metabolic syndrome is increasing worldwide (Misra et al., 2010 ▶). A hyperlipidemic conditionis associated with increased oxidative stress and overproduction of oxygen free radicals (Zalba et al., 2001 ▶). Some of lifestyle factors responsible for the development of metabolic diseases are described in the literature (e.g. such as physical inactivity and consumption of high-fat diet) (Talita et al., 2014 ▶; Nettleton and Katz, 2005 ▶). Diets rich in sucrose, dextrose, fructose, fat or any combination of these compounds make important changes in carbohydrate metabolism resulting in insulin resistance, type 2 diabetes, weight gain, adiposity, dyslipidemia and arterial hypertension in rodents (Kohli et al., 2010 ▶; Buettner et al., 2006 ▶; Kim et al, 2011 ▶). Hyperlipidemia following oxidative stress may cause oxidative modifications in low density lipoproteins, which play an important role in the progression of atherosclerosis (Parthasarthy et al., 1992 ▶). Several studies have shown that animals fed with high-fat diets are more likely to develop risk factors for metabolic disorder, such as hyperlipidemia (Li et al., 2010) hepatic steatosis (Pérez-Echarri et al., 2009 ▶) and oxidative stress (Hee and Seon, 2009 ▶). Metabolic syndrome exposes an individual to increased prevalence of venous thromboembolism (Ageno et al., 2006 ▶; Ay et al., 2007 ▶; Borch et al., 2010 ▶; Ray et al., 2007 ▶; Severinsen et al., 2009 ▶). Past studies have shown that dietary modifications such as low fat diets, high-fiber diets, diets rich in flavonoids and phenolic acids can reduce metabolic syndrome risk factors (Minich and Bland, 2008 ▶; Lyer et al., 2009 ▶). 

The modern anti-hyperlipidemic drugs like statins and synthetic antioxidants like probucol are widely used to treat atherosclerosis. Unfortunately, these drugs are not free of side effects (Lankin et al., 2003 ▶). To provide novel treatments for hyperlipidemia, it has been focused on the natural products that have very few side effects (Si-Yuan et al., 2013 ▶). The world ethnobotanical information reported a number of herbal medicines from the plants that are used for controlling hyperlipidemia and the related complications in patients (Dahanukar et al., 2000 ▶). Approximately 80% of the third world populations are almost entirely dependent on traditional medicines (Srinivasan, 2005 ▶). The phytochemical constituents of methanolic extract of *H. indicus* exhibited a great antioxidant activity (Visweswara et al., 2013 ▶). A study revealed that *H. indicus *potentially decreased serum cholesterol, triglyceride, free fatty acids and phospholipid (Sowmia and Kokilavani, 2007 ▶). High-fat diet induced hyperlipidemia in rats as characterized by decreased levels of antioxidant enzymes, increased levels of cholesterol profile and damages in hepatic tissues. However, a long-term consumption of high-fat diet that makes lipid changes could increase the possibility of metabolic function damage. In this regard, phenolic compounds of *H. indicus* have attracted scientists because of their strong *in vitro* and *in vivo* antioxidant activity (Tapiero et al., 2002 ▶; Rabia et al., 2015 ▶). Hence, the present study was doneto evaluate antioxidant and hypolipidemic activities of different extracts of *H. indicus *in rats fed with high-fat diet.

## Materials and Methods

**Collection and identification of the plant materials**

The roots of *H. indicus *(Linn.) were collected from Tirunelveli District, India. Botanical identification was carried out at the Botanical Survey of Medicinal Plants Unit, Siddha, Palayamkottai. Where, a voucher specimen No.MMC/2012/11 was deposited in the museum of the Department of Pharmacognosy, Madras Medical College, Chennai. The roots were dried under shade, segregated, pulverized by a mechanical grinder and passed through a 40 mesh sieve. The powdered plant materials were stored in an airtight container.

**Drugs and Chemicals**

Cholesterol was obtained from Central Drug House Ltd, Mumbai, India. Atorvastatin was purchased from Ranbaxy, India. Cholesterol kit was obtained from Span Diagnostics Ltd. Surat, India. All other chemicals were of analytical grade procured from reputed Indian manufacturers.

**Experimental animals**

The study was approved by the institutional animal ethical committee of Madras Medical College, Chennai. Male albino wistar rats weighing 135-160 g were obtained from animal facility and housed (six animals per cage at 25± 5°C). The relative humidity maintained between 55-58%. The animals were allowed to have free access to tap water and standard laboratory pellet *ad libitum*.

**Preparation of the extracts**

The powdered plant materials were successively extracted with petroleum ether (40-60°C), ethyl acetate (76-78°C) and methanol (80°C) by hot continuous percolation method in a Soxhlet apparatus (Harborne, 1984 ▶) for 24 hr. Extracts solvent was recovered under reduced pressure using rotary evaporator and subjected to freeze-drying in a lyophilizer until dry powder was obtained.

**Acute toxicity study**

Acute oral toxicity study was performed according to organization of economic cooperation and development (OECD) guidelines 423. Each group consisted of 3 rats and 5-2000 mg/kg of different extracts of *H. indicus* were used. All different extracts of *H. indicus *were suspended in 2% tween 80 and given to the rats via oral intubation. The animals were observed individually every 30 min after dosing the first 24hr thereafter daily for 14 days. The time at which signs of toxicity appear/disappear was observed systematically and recorded for each animal.

**Induction of hyperlipidemia and anti-hyperlipidemic activity**

Experimental hyperlipidemia was developed by feeding with a high-fat diet (Powdered Normal Chow, 365 g; lard, 310 g; casein, 250 g; cholesterol, 10 g; vitamin mix and mineral mix, 60 g; DL methionine, 0.3 g; yeast powder, 0.1 g; and NaCl, 0.1 g were mixed to prepare 1.0 kg of HFD). The high fat diet contained 5.33 kcal/g while the normal chow contained 3.80 kcal/g.

Thirty six rats were divided into 6 groups of six each. Group I served as normal control without any treatment. Group II served as HFD control. Animals of groups III, IV and V were administered HFD followed by different extracts of *H. indicus*. Group VI served as positive control and received atorvastatin (1.2 mg/kg body weight). The extracts as well as atorvastatin were suspended in 2% tween 80 (Satheesh and Kottai, 2012 ▶) and fed to the respective rats via oral intubation. At the end of study, all rats were euthanized by cervical dislocation after overnight fasting. Before euthanasia, blood was collected from the retro-orbital sinus plexus under mild ether anaesthesia. The blood samples were collected in heparinized tubes and plasma was separated. A liver tissue was separated for the cholesterol investigations. All the experimental activities were conducted according to the animal ethics committee recommendations.

**Histopathological study**

Liver slices were fixed in 10% formalin and embedded in paraffin wax. Sections of 5 µm thickness were prepared using a microtome and stained with haematoxylin- eosin. The sections were observed under microscope and photographs of each slide were taken at 40x magnification.

**Statistical analysis**

The data are presented as mean ± standard error mean (SEM) for six animals in each group. Statistical analysis of the data was performed using one-way analysis of variance (ANOVA) followed by Dunnett’s test. 

Differences between means were considered statistically significant if p<0.05.

## Results

**Extraction of plant material**

Phytochemical evaluation of the methanolic extract of *H. indicus *showed the presence of alkaloids, carbohydrates, glycosides, phenolic compounds, tannins, saponins glycoside, tannins, protein, amino acids and flavonoids constituents (Table 1). 

**Table 1:** Preliminary phytochemical study of different extracts of *Hemidesmus indicus*

**Sl. No**	**Tests**	**Pet. Ether extract**	**Ethyl acetate extract**	**Methanolic extract**
**1.**	Alkaloids	Negative	Positive	Positive
**2.**	Carbohydrates and glycosides	Negative	Negative	Positive
**3.**	Phenolic compounds and tannins	Negative	Negative	Positive
**4.**	Flavonoids	Negative	Negative	Positive

**Acute toxicity study**

After administration of 5 mg/kg, 50 mg/kg, 500 mg/kg and until 2000 mg/kg dose of different extracts of *H. indicus*, the animals didn’t show a behavioral abnormality, dyslipidemia, toxic or mortality in rats. Hence, *H. indicus* a dose of 200 mg/kg, p.o. was selected for further pharmacological investigations.

**Pharmacological interventions on oxidative stress and plasma lipid profile**

The high fat dietary treatment for 9 weeks caused a sustained increase in % body weight in rats. As shown in table 2, 3 and 4, HFD groups caused a significant decrease in CAT, SOD and GPX. Phenolic compounds may protect against oxidative damage. High fat diet rats have shown an abnormal increase in plasma total cholesterol, free cholesterol, ester cholesterol, phospholipids, triglycerides, atherogenic index, LDL, VLDL and decrease in HDL levels when, compared to rats fed with a standard diet (Table 5,6 and 7). The present study demonstrated that flavonoid increases the vasodilation response of cardiovascular disease patients (Vèronique and Christine, 2012 ▶) and various in vitro studies have shown antiplatelet activity (Ji et al., 2014 ▶).

**Table 2:** Effect of different extracts of *Hemidesmus indicus *on CAT (μ moles of H_2_O_2 _consumed min/mg/protein) activity of the aorta, heart and liver in rats

**Groups**	**Aorta**	**Heart**	**Liver**
**Control**	33.25±0.66	46.63±0.56	29.93±0.82
**High fat diet (HFD)**	21.50±0.40	29.22±0.55	15.29±0.54
**HFD + ** **petroleum ether extract of ** ***H. indicus*** ** 200mg/kg**	24.25±0.34	33.21±0.60	18.43±0.55
**HFD + ** **ethyl acetate extract of ** ***H. indicus*** ** 200mg/kg**	25.10±0.54	36.85±0.50	20.08±0.47
**HFD + methanolic extract of** *** H. indicus*** ** 200mg/kg**	30.64±0.60^ns^	44.94±0.67^ns^	28.70±0.81^ns^
**HFD + atorvastatin (1.2mg/kgbw)**	34.06±2.89^ns^	46.47±0.84^ns^	29.03±0.81^ns^

Values are expressed as mean ± SEM (n = 6). Data were analyzed using One-way analysis of variance followed by Dunnett’s multiple comparison test.

p< 0.001;

p< 0.05 considered significant; ns: non-significant; All groups are compared with normal control. CAT: catalase.

**Table 3:** Effect of different extracts of *Hemidesmus indicus *on SOD (unit min/mg/protein) activity of the aorta, heart and liver in rats

**Groups**	**Aorta**	**Heart**	**Liver**
**Control**	2.50±0.05	1.84±0.05	3.50±0.07
**High fat diet**	1.36±0.03	0.60±0.04	1.50±0.05
**HFD + PEEHI 200mg/kg**	1.62±0.01	0.68±0.05	1.86±0.06
**HFD + EAEHI 200mg/kg**	1.72±0.02	0.77±0.05	1.98±0.06
**HFD + MEHI 200mg/kg**	2.36±0.04^ns^	1.68±0.05^ns^	3.29±0.06^ns^
**HFD + atorvastatin (1.2 mg/kgbw)**	2.43±0.04^ns^	1.72±0.05^ns^	3.35±0.06^ns^

Values are expressed as mean ± SEM (n = 6). Data were analyzed using One-way analysis of variance followed by Dunnett’s multiple comparison test.

p<0.001;

p< 0.05 considered significant; ns: non-significant; All groups are compared with normal control. SOD: superoxide dismutase.

**Table 4:** Effect of different extracts of *Hemidesmus indicus *on GPX (mg of GSH consumed/min/mg protein) activity of aorta, heart and liver in rats

**Groups**	**Aorta**	**Heart**	**Liver**
**Control**	11.54±0.56	15.90±0.64	9.13±0.433
**High fat diet**	6.88±0.30	7.38±0.45	5.74±0.38
**HFD + PEEHI 200mg/kg**	8.00±0.25	9.09±0.52	6.39±0.39
**HFD + EAEHI 200mg/kg**	8.69±0.40	10.06±0.78	7.07±0.28
**HFD + MEHI 200mg/kg**	10.29±0.58^ns^	14.23±0.74^ns^	8.30±0.34^ns^
**HFD + atorvastatin (1.2 mg/kgbw)**	11.1±0.60^ns^	15.22±0.65^ns^	8.75±0.40^ns^

Values are expressed as mean ± SEM (n = 6). Data were analyzed using One-way analysis of variance followed by Dunnett’s multiple comparison test.

p<0.001,

p<0.01;

p<0.05 considered significant; ns: non-significant; All groups are compared with normal control.GPX: glutathione peroxidase.

**Table 5:** Effect of different extracts of *Hemidesmus indicus *on plasma lipid profile in control and experimental rats

**Groups**	**BW changes (gm)**	**TC (mg/dl)**	**FC (mg/dl)**	**EC (mg/dl)**
**I**	194.76±2.48	113.83±1.48	27.05±0.28	84.99±1.71
**II**	251.25±3.05	177.8±1.64	46.50±0.75	130.08±0.33
**III**	241.02±2.86	127.90±1.45	42.61±0.91	119.63±1.25
**IV**	238.80±2.71	127.46±1.49	42±0.89	118.81±1.38
**V**	204.51±2.47^ns^	119.63±1.69	28.95±0.35^ns^	87.00±1.78^ns^
**V**	200.53±2.50^ns^	116.51±1.26^ns^	28.09±0.21^ns^	85.73±1.80^ns^

Values are expressed as mean ± SEM (n = 6). Data were analyzed using One-way analysis of variance followed by Dunnett’s multiple comparison test.

p<0.001,

p<0.05 considered significant; ns: non-significant; All groups are compared with normal control. BW: body weight; TC: total cholesterol; FC: free cholesterol; EC: ester cholesterol.

**Table 6:** Effect of different extracts of *Hemidesmus indicus *on plasma lipid profile in control and experimental rats

**Groups**	**PLs (mg/dl)**	**TGs (mg/dl)**	**AI (mg/dl)**
**I**	106.53±0.41	78.54± 0.93	1.85±0.03
**II**	155.19±1.17	155.91±1.34	4.67±0.12
**III**	148.48±0.98	134.55±2.28	2.35±0.03
**IV**	147.74±1.14	116.41±1.98	2.28±0.03
**V**	110.09±0.50	84.19±1.24^ns^	1.95±0.03^ns^
**VI**	108.82±0.35^ns^	81.59±1.19^ns^	1.90±0.03^ns^

Values are expressed as mean ± SEM (n = 6). Data were analyzed using One-way analysis of variance followed by Dunnett’s multiple comparison test.

p<0.001,

p<0.05 considered significant; ns: non- significant. All groups are compared with normal control. PLs: phospholipids; TGs: triglycerides; AI: atherogenic index.

**Table 7:** Effect of different extracts of *Hemidesmus indicus *on plasma lipoprotein in control and experimental rats

**Groups**	**HDL (mg/dl)**	**LDL (mg/dl)**	**VLDL (mg/dl)**
**I**	58.87±0.63	39.29±0.61	17.50±0.48
**II**	24.98±1.23	104.91±1.95	35.67±1.31
**III**	29.43±1.27	94.50±2.34	32.66±1.04
**IV**	35.76±1.18	84.01±2.37	32.41±1.18
**V**	53.84±0.92	46.26±1.04	19.94±0.43^ns^
**VI**	55.77±1.18^ns^	41.99±1.13^ns^	19.14±0.44^ns^

Values are expressed as mean ± SEM (n = 6). Data were analyzed using One-way analysis of variance followed by Dunnett’s multiple comparison test.

p<0.001,

p<0.05 considered as significant; ns: non significant; All groups are compared with normal control.HDL: high density lipoprotein; LDL: low density lipoprotein; VLDL: very low-density lipoprotein.

After treatment with 200 mg/kg of methanolic extract of *H. indicus* and atorvastatin 1.2 mg/kg, a significant response against high-fat diet induced body weight, oxidative stress and hyperlipidemia was observed. The present study demonstrated that flavonoids increase the vasodilation response in cardiovascular disease patients (Vèronique and Christine, 2012 ▶) and various *in vitro* studies have shown anti-platelet activity for flavonoids (Ji et al., 2014 ▶). 

**The effects of various treatments on histology of the liver**

**Figure 1:**
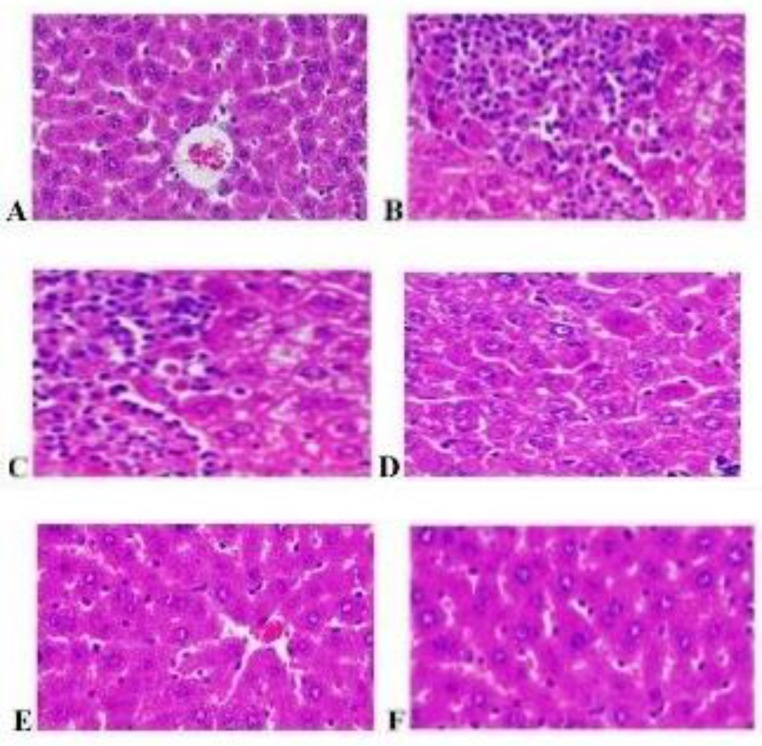
Effect of various treatments on histology of liver tissue

High-fat diet-treated rats produced significant changes in hepatic tissue architecture such as micro and macro vascular steatosis, increased fatty infiltration, inflammation (over activation of kupffer cells), sinusoidal dilation, degeneration of central vein and vacuolization, as compared to normal liver histology. Treatment with *H.indicus *200 mg/kg significantly attenuated these effects of high fat diet, as compared to HFD control (Figure 1).

A: Normal control; B: High-fat diet; C: High-fat diet with petroleum ether extract of *H. indicus* 200 mg/ kg bw; D: High-fat diet with ethyl acetate extract of *H. indicus* 200 mg/kg bw; E: High-fat diet with methanolic extract of *H. indicus* 200 mg/kg bw; F: High-fat diet with standard drug of atorvastatin (1.2 mg/kg bw)

## Discussion

The present investigation was undertaken to assess the anti-oxidant and antihyperlipidemic activity of *H. indicus*. A previous report demonstrated that flavonoid rich in tea that increases the vasodilation response of cardiovascular disease patients (Vèronique and Christine, 2012 ▶) and various *in vitro* studies have shown anti-platelet activity of flavonoids. (Ji et al., 2014 ▶). Several epidemiological studies have shown flavonoid intake is associated with a low risk of cardiovascular disease (Marjorie et al., 2012 ▶). Our results indicated that the phytochemical constituents of MEHI may play an important role in its antioxidant and anti-hyperlipidemic activity.

Additional research is warranted on flavonoid and cardiovascular disease prevention and survival, since several flavonoids including the anthocyanins, flavones, flavan-3-ols and proanthocyanidins may have blood pressure lowering effects and may have beneficial effects on other cardiovascular disease risk factors as well (Phang et al., 2011 ▶).

In acute toxicity studies, *H. indicus *up to 2000 mg/kg) was found to be non-toxic and did not cause death among the tested animals. Previous studies reported that polyphenolic compounds may protect against oxidative damage (Simonyi et al., 2010 ▶). Marisol et al. reported that modulation of nitric oxide (NO) availability plays an important role in ischemic stroke (Marisol et al., 2013 ▶). In the present investigation, we showed that HFD-treated rats had significant oxidative stress in terms of CAT, SOD, GPX levels. As shown in tables 5, 6 and 7, average body weight, TC, FC, EC, PLs, TGs, AI, HDL, LDL and VLDL were increased. Furukawa et al. (Furukawa et al., 2004 ▶) found that oxidative stress is highly correlated with a wide variety of inflammatory and metabolic disease states including obesity. Moreover, Mishra (Mishra, 2004 ▶) showed that free radicals may adversely affect cell survival following membrane damage through the oxidative damage of lipid, protein and irreversible DNA modification. Abdominal obesity and insulin resistance were proposed as the main causal factors of metabolic syndrome (Christian et al., 2013 ▶). 

Consistently, we found that MEHI increased the level of SOD, CAT, and GPX, decreased body weight and plasma lipid profile. Manju (Manju et al., 2010 ▶) demonstrated that oxidative damage is aggravated by the decrease in antioxidant enzyme activities such as superoxide dismutase, catalase, glutathione S-transferase and glutathione peroxidase which act as free radical scavengers in conditions associated with oxidative stress. Here, we examined the effect of MEHI on dyslipidemia and increased SOD, CAT and GPX activity of the aorta, heart, and liver in rats. 

HFD-treated rats significantly (p<0.001) increased body weight. Also, a previous study revealed that obesity and hypercholesterol were associated with defective thermogenesis (Colin et al., 2005 ▶). More importantly, our study demonstrated that a potential weight-reducing effect for MEHI. We found that high-fat diet-treated rats show increasing levels of plasma total cholesterol which is consistent with earlier studies (Vijaimohan et al., 2006 ▶; Mehta et al., 2003 ▶). After treatment with MEHI, it possibly decreases total cholesterol and may reduce the risk of heart attacks, strokes and death. 

Consumption of high-fat diet is crucial for the development of free and ester cholesterol (Table 5). This condition of high cholesterol may initiate atherosclerosis (Jeng-Jiann and Shu, 2011 ▶). In the present study, we investigated the effect of MEHI on the high-fat diet-treated rats and results indicated that the lipid lowering effect may due to the inhibition of hepatic cholesterogenesis (Nishant et al., 2011 ▶). Moreover, the rats that received MEHI 200 mg/kg exhibited reduction in phospholipids (PLs) and triglycerides (TGs) level. It was reported that high-fat diet-treated rats showed a significant increase in the level of plasma triglycerides due to decrease in the activity of lipoprotein lipase (Satheesh and Kottai, 2012 ▶). 

Kumar et al. (Kumar et al., 2015 ▶) studied atherogenic index which indicates the deposition of foam cells or plaque or fatty infiltration or lipids in the heart, coronaries, aorta, liver, kidney which increases the risk of oxidative damage in the above organs. As shown in Table 7, MEHI-treated rats demonstrated an increase in the HDL level as compared to high-fat diet-treated rats. Arshag (Arshag, 2009 ▶) study revealed low HDL-cholesterol considered the most significant risk factor for atherosclerosis. Furthermore, Donovan et al. (Donovan et al., 2011 ▶) have shown that increase in the concentration of HDL-cholesterol reduces the morbidity and mortality rate in cardiovascular patients. Keevil (Keevil et al., 2007 ▶) studied high-fat diet to elevate LDL and VLDL-cholesterol. It may increase the risk for hyperlipidemia and cardiovascular diseases. Treatment with MEHI markedly reduced plasma LDL and significantly reduced VLDL. Our findings also showed that elevated LDL cholesterol is one of the causes of coronary heart diseases (Antonio and Jr, 2011 ▶). 

Sung (Sung et al., 2014 ▶) reported that hepatic steatosis is a common consequence of obesity, and its prevalence (Amanda et al., 2011 ▶) has been further characterized with hepatic fat accumulation. After treatment with MEHI reversed the effect of high-fat diet-induced liver damage (Figure 1). Our findings demonstrated tissue architecture destroy due to chronic high-fat diet intake which was effectively prevented by MEHI. This effect may be due to the cellular migration to injured sites and accumulation of collagen mucopolysaccharides.

## Note

*Please cite this paper as: *

Suganya V, Vetriselvan S, Velmurugan C, Sarath C. Anti-oxidant and Anti-hyperlipidemic activity of *Hemidesmus indicus *in rats fed with high-fat diet.Avicenna J Phytomed, 2016; 6 (5): 516-525.
